# Psychological characteristics of athletes on the Vietnamese national weightlifting team prior to the 2019 Asian Championships: a cross-sectional descriptive study

**DOI:** 10.3389/fspor.2026.1945226

**Published:** 2026-10-07

**Authors:** Ngoc Quang Dinh

**Affiliations:** Institute of Sport Sicence, Technology and Foreign Affairs, Bac Ninh Sport University of Vietnam, Bắc Ninh, Vietnam

**Keywords:** competitive anxiety, emotional states, national team, psychological characteristics, psychological reliability, psychological state, Vietnam, weightlifting athletes

## Abstract

This cross-sectional descriptive study was conducted to evaluate the psychological characteristics of athletes in the Vietnamese National Weightlifting Team prior to the 2019 Asian Championships. The study participants included 17 elite athletes (9 males and 8 females) from the Vietnamese National Weightlifting Team. Standardized psychometric and specialized assessment instruments—including the Competitive Anxiety Inventory (CAH), Profile of Emotional States (PET), Sport Competition Anxiety Test (SCAT), and Psychological Reliability Assessment tests—were administered to assess health status, emotional states, competitive anxiety, and psychological stability. Given the cross-sectional nature of the dataset, the findings reflect specific pre-competition psychological states during that historical training macrocycle rather than a real-time profile of the current roster. The results indicated that the participants exhibited varying levels of pre-competition psychological activation; while general health status and positive emotional indicators showed stable baseline parameters, specific markers of competitive anxiety and emotional regulation varied among the athletes. Due to the limited sample size (n=17), sex-specific subgroup analyses yielded no statistically significant differences in primary anxiety and emotional variables (p>0.05). These insights highlight the necessity of individualized psychological monitoring in elite weightlifting and provide a foundational baseline and methodological reference point for future longitudinal and cross-national investigations in strength sports.

## 1 Introduction

Weightlifting is a sport that requires a harmonious combination of physical strength, technical-tactical proficiency, and the psychological state of the athletes. In the context of modern sports, as the technical and tactical levels of athletes are increasingly refined and physical conditioning is intensively enhanced, psychological factors are considered to play a pivotal, decisive role in competitive performance and serve as one of the core elements driving fierce competition in weightlifting. According to Hanton et al. (1) and Mellalieu et al. (2), success is determined not only by physiological adaptations, biomechanical proficiency, and technical mastery, but also by psychological resilience and optimal emotional regulation under high-stakes competitive pressure. Anshel et al. (3) and Weinberg & Gould (4) noted that weightlifting is an explosive sport characterized by single-effort execution that demands maximal neuromuscular recruitment within milliseconds, rendering athletes exceptionally vulnerable to pre-competition anxiety, emotional fluctuations, and psychological instability. While extensive sport psychology literature has examined endurance and team sports, empirical investigations focusing specifically on weightlifting athletes—particularly national-team cohorts—remain relatively limited, especially within developing sporting systems in Southeast Asia.

Understanding the psychological profiles of national-team athletes is critical for sport scientists and coaches aiming to design targeted psychological interventions, prevent overtraining, and enhance performance consistency (5, 6, 17). Previous ministerial and institutional studies in Vietnam have historically prioritized physiological and nutritional tracking, often overlooking the systematic assessment of psychological indicators such as competitive anxiety, emotional states, and psychological reliability. Consequently, there is a distinct lack of peer-reviewed empirical data mapping the psychological characteristics of Vietnamese national-team weightlifters during major international preparation cycles (7, 8).

Furthermore, existing literature frequently suffers from a lack of transparency regarding context-specific psychometric applications and fails to delineate baseline psychological markers across competitive sexes within weight-class sports. Addressing this gap is vital not only for retrospective evaluation but also for establishing methodological frameworks for future longitudinal tracking in strength sports.

Therefore, this cross-sectional descriptive study aimed to evaluate the specific psychological characteristics—namely health status, emotional states, competitive anxiety, and psychological reliability—of athletes in the Vietnamese National Weightlifting Team prior to the 2019 Asian Championships. By providing a transparent, methodologically rigorous examination of this unique cohort, this study seeks to contribute foundational data to the international literature on sport psychology in weightlifting, guide future psychological training and adjustment protocols for national-team athletes, and ultimately enhance training efficiency, maximize athletic potential, and contribute to the sustainable development of Vietnamese weightlifting on the international stage (9, 10).

## 2 Research methodology

### 2.1 Survey subjects

Participants in this cross-sectional descriptive study consisted of 17 athletes from the Vietnamese National Weightlifting Team, including 8 females and 9 males. The survey was conducted in alignment with the team's training macrocycle, scheduled prior to the 2019 Asian Championships (8).

The eligibility criteria for participant selection were established as follows:

1. Being an official member of the Vietnamese National Weightlifting Team during the 2019 training phase;
2. Actively participating in the centralized training program preparing for major international competitions; and
3. Having full cognitive capacity and willingness to complete the psychometric assessment battery used by the research team.

The sample size was limited (*n* = 17; 9 males, 8 females) because this cohort represented the complete official roster of the Vietnamese National Weightlifting Team assembled for centralized training at the time of the study. Given the highly specialized nature and restricted population size of elite national-team athletes, evaluations of this entire cohort provide crucial baseline insights, aligning with standard methodological practices in elite sports science literature where access to national-team cohorts is inherently constrained.

### 2.2 Data collection method

During the research process, the research team used psychological testing methods to collect relevant data to determine the psychological characteristics of weightlifting athletes in the Vietnamese national team (7, 20). To meet specific requirements for methodological transparency, the origin, standardization, and reliability parameters of each testing instrument are detailed below:

1. Competitive Anxiety Inventory (CAH) test: Developed based on the multidimensional anxiety framework (11) with verified internal consistency and established Cronbach’s alpha values ranging from 0.78 to 0.86. The CAH test questionnaire consists of 3 groups of questions assessing Health Status, Positivity and Mood, with each group containing 10 questions. Each question is scored from a minimum of 1 point to a maximum of 7 points. The results are classified into 3 levels based on standardized scoring thresholds: Good (5.01–7 points); Average (3.01–5 points); Poor (1–3 points).
2. Profile of Emotional States (PET Test) (T = 32 × 6): Adapted from established sports-specific emotional frameworks (such as adaptations of the Profile of Mood States—POMS (12). The PET Test questionnaire consists of 4 groups of questions to determine the pre-competition emotions of athletes, covering 4 areas: Anxiety about personal failure; Self-confidence; Anxiety from social expectations; and Somatic anxiety. The PET test evaluates immediate emotional states and moods using standardized scoring distributions, with internal consistency coefficients for the subscales in sport-specific applications routinely exceeding 0.70. Each group consists of 8 questions, with each question scored from a minimum of 1 point to a maximum of 6 points. Results are classified into 3 levels: High (34.61–48.0 points); Average (21.31–34.6 points); Low (8–21.3 points).
3. SCAT Test: Developed by Martens and colleagues (11, 19). The SCAT has been demonstrated to have high reliability, with internal consistency (KR−20) ranging from 0.95 to 0.97 and test-retest reliability of r=0.85. The SCAT Test consists of 15 questions, of which 10 are scored to assess the levels of competitive anxiety in athletes. Each question is scored from a minimum of 1 point to a maximum of 3 points. The total score ranges from a minimum of 10 to a maximum of 30 points. Results are classified into 3 levels: High (23.35–30.0 points); Average (16.68–23.34 points); Low (10–16.67 points).
4. Psychological reliability assessment test: Developed from the classical sport psychology and psychodiagnostic frameworks established by V.E. Milman (13) and V.L. Marischuk (14) to evaluate operational reliability and stress tolerance in elite athletes. The questionnaire for this test consists of 22 questions, allowing for the assessment of psychological reliability based on: Emotional stability in competition, Emotional regulation ability, Resistance to interference, Motivation and willpower, Nature of emotional response (Increased, Neutral, Decreased). The specific evaluation criteria are as follows: A score of “0” corresponds to an average level of psychological reliability; a score with a “-” sign corresponds to a decrease in psychological reliability; a score with a “+” sign corresponds to an increase in psychological reliability. The nature of emotional response has 3 levels: Aroused (Increased); Neutral; Decreased (7). While these diagnostic methods have been widely applied in structured training monitoring systems, their interpretation relies on established practical evaluation norms rather than broad contemporary Western psychometric scales, serving as valuable localized baseline indicators of competitive stability.

The psychological state assessment tools mentioned above were administered entirely in Vietnamese. The tests have undergone a process of translation, cross-cultural adaptation, and linguistic standardization to ensure semantic and psychological equivalence for elite athletes in Vietnam. The results of reliability testing for the scales achieved a high level of stability (with internal consistency and test-retest reliability coefficients falling within acceptable limits), meeting the requirements for psychological measurement in high-performance sports.

### 2.3 Implementation process

The research team coordinated with the coaching staff of the Vietnamese national weightlifting team to organize a meeting to discuss and provide detailed instructions to the athletes on how to answer the questionnaire forms for each psychological test. Afterward, the questionnaires were distributed to each athlete, who individually studied and completed them according to each test form. The distribution and collection of the questionnaires were conducted simultaneously within a timeframe of 48 to 72 h prior to the official competition. The research team collected the completed questionnaires, compiled the data and determined the test results based on the evaluation standards established above. On that basis, the analysis and assessment of the test results were conducted (8).

### 2.4 Mathematical and statistical methods

The data collected from the psychological test results for each athlete through the 04 tests were compiled and calculated according to the specific formula of each test to determine the score level for each content, which served as a basis for identifying the psychological characteristics of each athlete. All data processing, computations, and comparative analyses were performed using standardized spreadsheet software (Microsoft Excel) utilizing built-in mathematical and statistical functions. Descriptive statistics were calculated and presented as means (x¯) and standard deviations (*δ*) for continuous variables, and as absolute frequencies and percentages (%) for categorical variables. To evaluate the differences between male and female athletes, independent-samples t-tests (t) were applied to compare group means based on standard parametric algorithms. Given the complete-cohort design of the national team squad (*n* = 17), these comparative evaluations provide transparent baseline measures of gender-based variations in psychological parameters. The threshold for statistical significance was set at the 5% level (*P* < 0.05) (7). In addition to independent-samples t-tests, prior to analysis, the homogeneity of variance of the data was evaluated using Levene's test, while the normality of distribution was preliminarily checked via frequency histograms and skewness coefficients, confirming that the data did not severely violate parametric assumptions. Due to the limited sample size (*n* = 17), alongside conventional *p* values, Hedges' g effect sizes and 95% confidence intervals (95% CI) were also calculated and examined to accurately quantify the magnitude of practical differences and enhance reliability when interpreting psychological variations by sex.

## 3 Results and discussion

The results of the survey and assessment of the current state of psychological characteristics of athletes in the Vietnamese National Weightlifting Team are presented in Table 1.

**Table 1:** Characteristics of the psychological state of the athletes on the Vietnamese national weightlifting team prior to the 2019 Asian championships (*n* = 17).

No.	Psychological factor			Male athletes (*n* = 9)(x¯±*δ*)	Female athletes (*n* = 8)(x¯±*δ*)	Comparison			
						t	P	Hedges’ g	95% CI
1	Health status (point)			5.30 ± 0.49	5.88 ± 0.41	2.52	≤0.05	−1.21	[−2.25, −0.17]
2	Positivity (point)			5.20 ± 0.47	5.78 ± 0.72	1.87	>0.05	−0.91	[−1.92, 0.10]
3	Mood (point)			5.58 ± 0.72	5.93 ± 0.44	1.15	>0.05	−0.56	[−1.53, 0.42]
4	Anxiety state	Anxiety about personal failure (point)		26.56 ± 9.26	26.50 ± 7.29	0.01	>0.05	0.01	[−0.96, 0.98]
		Self-confidence (point)		33.78 ± 12.50	36.00 ± 4.21	0.47	>0.05	−0.23	[−1.20, 0.74]
		Anticipatory anxiety (point)		25.67 ± 9.90	22.25 ± 7.29	0.77	>0.05	0.38	[−0.61, 1.36]
		Somatic anxiety (point)		23.78 ± 7.63	17.38 ± 4.47	2.03	>0.05	0.99	[−0.04, 2.01]
		Competitive anxiety (point)		19.11 ± 2.71	19.13 ± 2.59	0.01	>0.05	−0.01	[−0.98, 0.96]
5	Psychological reliability assessment	Emotional stability in competition (point)		−1.04 ± 0.48	−1.02 ± 0.31	0.08	>0.05	−0.05	[−1.02, 0.92]
		Emotional regulation ability (point)		−0.39 ± 0.48	−0.50 ± 0.42	0.49	>0.05	0.24	[−0.73, 1.21]
		Resistance to interference (point)		−0.07 ± 0.85	−0.21 ± 0.71	0.34	>0.05	0.17	[−0.80, 1.14]
		Motivation and willpower (point)		0.54 ± 0.45	0.38 ± 0.33	0.82	>0.05	0.40	[−0.59, 1.39]
		Nature of emotional response	Decreased	2 athletes (22.22%)	3 athletes (37.50%)	-	-	-	-
			Neutral	7 athletes (77.78%)	5 athletes (62.50%)	-	-	-	-
			Aroused	0 athletes (0.00%)	0 athletes (0.00%)	-	-	-	-

The results presented in Table 1 provide a comprehensive overview of the psychological state characteristics of elite Vietnamese national weightlifters prior to the 2019 Asian Championships. The evaluation scores for Health Status, Positivity, and Mood among both male and female athletes generally remained at favorable levels (ranging from 5.20 to 5.88 points). Notably, female athletes achieved higher scores across these dimensions, with Health Status showing a statistically significant difference [5.88 ± 0.41 for females versus 5.30 ± 0.49 for males, *p* <= 0.05, Hedges' g = −1.21, 95% CI (−2.25, −0.17)]. This finding aligns with recent sport psychology investigations regarding gender-specific psychological responses in elite sports [e.g., Nicholls, Polman, & Levy (15)], indicating that female competitors tend to exhibit a more structured self-awareness regarding bodily recovery signals and adhere more effectively to recovery protocols during high-stress pre-competition tapering periods. However, due to the limited survey sample size, the interpretation of these observations should be cautious, primarily acknowledging preliminary descriptive trends regarding the ability to receive physical recovery cues during the pre-competition preparation phase of the athletes. Furthermore, the stability observed in Mood and Health Status reflects an adaptive capacity to cope with major stressors, consistent with findings from elite athletic state management studies. Conversely, the high proportion of athletes exhibiting moderate scores in Positivity (such as male athletes at 5.20 ± 0.47) uncovers an underlying vulnerability: despite a high perception of general health, sustaining unwavering psychological positivity and resilience under continental-level competitive pressure remains a challenge that necessitates scientific, targeted psychological training and regulation (21).

Regarding anxiety states, both male and female weightlifters predominantly exhibited moderate levels across sub-dimensions, in which across most anxiety states, the evaluation scores of anxiety levels in male athletes tended to be higher compared to females. Conversely, the evaluation scores of self-confidence states in female athletes also tended to be higher than in male athletes. Although the comparison results of evaluation scores between male and female weightlifters did not reach statistical significance (*p* > 0.05), however, this descriptive mean discrepancy [for instance, the somatic anxiety index recorded a difference with Hedges' g = 0.99, with the 95% confidence interval being (−0.04, 2.01)] has also demonstrated certain different preliminary descriptive trends of psychological manifestations between the groups of male and female athletes when facing psychological pressures during the pre-competition stage. Weightlifting is an explosive strength discipline where success is determined by split-second execution and precise motor control, therefore, if unmanaged, the aforementioned psychological issues will often manifest externally as physical tension or fatigue (18). These survey results reinforce the theoretical framework of competitive anxiety and stress appraisal in elite sports established by Hanton, Mellalieu, and Fletcher (1), alongside Nicholls et al. (15), regarding multidimensional anxiety structures in weight-class and explosive sports.

Regarding psychological reliability, a striking dichotomy emerged from the empirical data. While athletes' ratings for Drive and Willpower remained positive (0.54 ± 0.45 for males and 0.38 ± 0.33 for females), indicators reflecting emotional control capacity skewed negatively. Specifically, Competitive Emotional Stability averaged −1.04 ± 0.48 for males and −1.02 ± 0.31 for females; Emotional Regulation Ability averaged −0.39 ± 0.48 for males and −0.50 ± 0.42 for females; and Resistance to Interference averaged −0.07 ± 0.85 for males and −0.21 ± 0.71 for females. This uncovers a critical practical implication: while elite weightlifters possess a robust internal drive and ambition for success, their capacity to safeguard emotional stability against competitive stressors exhibits notable vulnerabilities. To address this limitation within high-performance environments, modern psychological skills training and emotional regulation frameworks must be systematically integrated (following Weinberg & Gould, 2019) (4). This finding also provides direct empirical support for the implementation framework of the ministerial-level scientific project directed by the lead author (2021) concerning the application of psychological adjustment methods for national athletes, confirming that psychological coaching must prioritize emotional regulation alongside motivational reinforcement.

To further clarify the assessment results of the psychological state of male and female weightlifting athletes on the national team, the research team proceeded to summarize the psychological classification results for each factor. The results are presented in Tables 2–4.

**Table 2:** Health status, activation, and mood of the athletes on the Vietnamese national weightlifting team prior to the 2019 Asian championships (*n* = 17).

Gender	Classification	Results					
		Health status		Positivity		Mood	
		Quantity	%	Quantity	%	Quantity	%
Male (*n* = 9)	Good	7	77.78	4	44.44	7	77.78
	Average	2	22.22	5	55.56	2	22.22
	Poor	0	0.00	0	0.00	0	0.00
Female (*n* = 8)	Good	8	100.00	6	75.00	7	87.50
	Average	0	0.00	2	25.00	1	12.50
	Poor	0	0.00	0	0.00	0	0.00
Total (*n* = 17)	Good	15	88.24	10	58.82	14	82.35
	Average	2	11.76	7	41.18	3	17.65
	Poor	0	0.00	0	0.00	0	0.00

**Table 3:** Characteristics of anxiety states of the athletes on the Vietnamese national weightlifting team prior to the 2019 Asian championships (*n* = 17).

Gender	Classification	Result									
		Anxiety about personal failure		Self-confidence		Anticipatory anxiety		Somatic anxiety		Competitive anxiety	
		Quantity	%	Quantity	%	Quantity	%	Quantity	%	Quantity	%
Male (*n* = 9)	High	2	22.22	5	55.56	3	33.33	1	11.11	0	0.00
	Average	3	33.33	2	22.22	4	44.44	4	44.44	8	88.89
	Low	4	44.44	2	22.22	2	22.22	4	44.44	1	11.11
Female (*n* = 8)	High	1	12.50	5	62.50	0	0.00	0	0.00	1	12.50
	Average	5	62.50	3	37.50	3	37.50	1	12.50	5	62.50
	Low	2	25.00	0	0.00	5	62.50	7	87.50	2	25.00
Total (*n* = 17)	High	3	17.65	10	58.82	3	17.65	1	5.88	1	5.88
	Average	8	47.06	5	29.41	7	41.18	5	29.41	13	76.47
	Low	6	35.29	2	11.76	7	41.18	11	64.71	3	17.65

**Table 4:** Results of psychological test reliability evaluation for the athletes on the Vietnamese national weightlifting team prior to the 2019 Asian championships (*n* = 17).

Gender	Classification	Results							
		Emotional stability in competition		Emotional regulation		Resistance to interference		Motivation and willpower	
		Quantity	%	Quantity	%	Quantity	%	Quantity	%
Male (*n* = 9)	Increased	0	0.00	1	11.11	5	55.56	8	88.89
	Average	0	0.00	2	22.22	0	0.00	0	0.00
	Decreased	9	100	6	66.67	4	44.44	1	11.11
Female (*n* = 8)	Increased	0	0.00	1	12.50	3	37.50	7	87.50
	Average	0	0.00	0	0.00	0	0.00	0	0.00
	Decreased	8	100	7	87.50	5	62.50	1	12.50
Total (*n* = 17)	Increased	0	0.00	2	11.76	8	47.06	15	88.24
	Average	0	0.00	2	11.76	0	0.00	0	0.00
	Decreased	17	100	13	76.47	9	52.94	2	11.76

“Increased” and “Decreased” denote categorical states relative to normative mean values (cross-sectional data), not longitudinal changes over time.

The detailed classification results obtained in Table 2 continue to shed light on the distribution structure of baseline psychological states among elite Vietnamese national weightlifters prior to the tournament, thereby reflecting the psycho-physiological adaptation levels of both male and female athletes. Specifically, the majority of athletes maintained their Health Status at a Good level, accounting for a high proportion of 88.24% of the entire cohort. Notably, 100% of female athletes achieved a Good rating, whereas this rate among male athletes was only 77.78%, with 22.22% falling into the Average category and no cases recorded in the Poor category. This distribution further reinforces prior observations regarding female athletes' capacity for self-awareness and adherence to physical recovery protocols during the pre-competition tapering period. This aligns closely with research on stress appraisal and psychological adaptation by Nicholls, Polman, and Levy (15).

However, a critical and noteworthy finding emerges from the evaluation of Positivity: although 58.82% achieved a Good level, as many as 41.18% of the total athletes attained only an Average level. This trend is more pronounced among male athletes, with only 44.44% reaching a Good level while up to 55.56% remained at the Average level. Meanwhile, the data indicate that female athletes tend to exhibit a higher proportion of achieving a Good level compared to male athletes (at 75.00%). As is well known, weightlifting is an explosive sport demanding high neural arousal and absolute confidence in executing maximal loads within a short timeframe. Consequently, the fact that nearly half of the male athletes maintained only an average level of positivity reveals a crucial psychological blind spot. This also proves that, alongside a good physical condition, maintaining an optimistic, active, and steadfast mental state under high continental competitive pressure remains a significant challenge for male athletes of the Vietnamese national weightlifting team, requiring managers and coaches to pay attention to providing guidance in selecting appropriate training and psychological adjustment plans to further improve for the athletes.

Regarding Mood, the majority of athletes maintained positive stability, with 82.35% achieving a Good rating (including 77.78% for males and 87.50% for females) and only 17.65% at an Average level. This high degree of similarity in mood factors indicates a relatively stable nervous system prior to international competition, reflecting the overall effectiveness of training management. Simultaneously, it serves as a crucial foundation for coaches and sport psychologists to formulate specialized emotional regulation strategies aimed at optimizing peak performance timing for each athlete.

The detailed evaluation results regarding the anxiety states of Vietnamese national weightlifting athletes prior to the 2019 Asian Championships in Table 3 reveal a notable observation: the majority of athletes recorded low anxiety levels, specifically in Somatic Anxiety (accounting for 64.71%), Anticipatory Anxiety (41.18%), and Anxiety about Personal Failure (35.29%), accompanied by a predominant proportion exhibiting high Self-confidence (58.82%). However, a deeper analysis of the distribution ranges reveals a critical psychological concern: the proportion of athletes maintaining moderate anxiety states spans a wide range from 29.41% to 76.47%, with Competitive Anxiety standing out as 76.47% of athletes were assessed only at the average level. Such fluctuations at moderate anxiety levels indicate that the nervous systems of weightlifting athletes had not achieved an optimal state at the time of testing and remained vulnerable under performance pressure. This is a particularly noteworthy issue, given that weightlifting is an explosive sport requiring maximal force output within a fraction of a second, where microscopic technical errors lead to failure.

Furthermore, the data indicates that a subset of athletes still suffers from high anxiety levels, concentrated primarily on factors such as Anxiety about Personal Failure and Anticipatory Anxiety (both accounting for 17.65%). These manifestations reflect heavy psychological pressure regarding performance expectations, the fear of disappointing the coaching staff, or being surpassed by competitors. If left unaddressed or unmanaged, this cognitive pressure may manifest as negative physiological phenomena such as muscle stiffness and the loss of motor coordination rhythm. This argument aligns thoroughly with the theoretical frameworks of multidimensional anxiety structures and stress appraisal in elite sports established by Hanton, Mellalieu, and Fletcher (1), as well as empirical studies on coping strategies by Nicholls, Polman, and Levy (15).

When comparing the assessment results between male and female athletes, it can be seen that across most anxiety-level evaluation indices, male athletes record a predominantly higher percentage of occurrence in high anxiety states compared to female athletes. Although constrained by the small sub-sample size, this descriptive discrepancy suggests a preliminary trend that national weightlifting male athletes may face greater qualitative pressure and specific psychological burdens, thereby demanding that psychological skills training be highly individualized, prioritizing methods and techniques for monitoring and regulating anxiety and emotional states prior to major competitions (22).

The evaluation results of psychological reliability in Table 4 indicate several existing issues within the competitive psychological structure of Vietnamese national weightlifting athletes prior to the 2019 Asian Weightlifting Championships. Specifically, indices concerning Emotional Stability in Competition, Emotional Regulation Ability, and Resistance to Interference predominantly recorded results at the decreased level. Notably, for Emotional Stability in Competition, 100% of the participating athletes fell into the decreased category. This presents certain disadvantages for the athletes, as deficiencies in emotional stability, coupled with limitations in emotional regulation and interference-coping capacities, act as detrimental factors affecting motor coordination rhythms, thereby reducing the execution efficiency of technical movements and competitive performance (23).

In stark contrast to the emotional stability and control indices, the Motivation and Willpower index demonstrated highly positive signals, with up to 88.24% of athletes reaching the increased level. This pronounced contrast indicates that national weightlifting athletes consistently possess a strong desire to contribute, high determination, and robust competitive drive. However, when such intense aspirations and expectations are coupled with deficiencies in emotional control and resistance to external interference, they become the underlying cause of negative feedback loops, amplifying neural tension and triggering drastic emotional state fluctuations throughout the competition process. These findings align with theoretical frameworks on psychological reliability and mental toughness in sports established by Gould et al. (16), as well as empirical studies on emotional control under pressure by Jones (5).

When comparing the classification results of psychological reliability between male and female athletes, it is observed that the decline in competitive emotional stability occurs universally across all (100%) male and female athletes of the Vietnamese national weightlifting team. Furthermore, regarding the emotional regulation ability index, female athletes exhibited a higher rate of decline compared to males (87.50% versus 66.67%). This reality necessitates that sports psychology training programs incorporate intensive drills simulating real competitive pressures to cultivate athletes' resistance to interference and self-regulation capacities, thereby optimizing psychological reliability prior to major tournaments.

## 4 Conclusion

This study has identified the psychological characteristics and recorded several group-specific manifestation trends among the athletes of the Vietnamese national weightlifting team prior to the 2019 Asian Championships. The research findings indicate that athletes maintain health status, positivity, mood, and motivation-willpower at good levels; the majority exhibit low levels of somatic anxiety. However, a notable proportion of athletes experience moderate levels of competitive anxiety, personal anxiety, and anticipatory anxiety. Concurrently, very high proportions of athletes suffer from declines in emotional stability (100%), emotional regulation ability (76.47%), and resistance to interference (52.94%). These limitations pose a risk of creating reverse feedback pressure, impairing the rhythm of technical coordination at critical moments. Therefore, the results of this study suggest that in the preparation process for national weightlifting athletes, the coaching staff should pay attention to orienting training and creating appropriate psychological simulation environments to support athletes in optimizing psychological stability and enhancing competitive performance.

## 5 Limitations and generalizability

This study has several limitations that need to be transparently acknowledged, specifically as follows:

Small sample size: The sample size (n=17; comprising 9 males and 8 females) reflects the entire limited total population of the national weightlifting team at the time of the survey, resulting in limited statistical power for subgroup analyses by gender.

Study design and data nature: The cross-sectional descriptive research design, combined with the self-reported nature of the psychological measurement tools, only allows for capturing the state at a strategic time point right before the competition (2019 Asian Weightlifting Championships) rather than establishing causal relationships or longitudinal variations.

Time gap and personnel fluctuations: The time elapsed from data collection to publication is historical; combined with the specific characteristics of personnel turnover among athletes and coaching staff across cycles, these findings reflect a foundational picture of a distinct and critical phase.

Cross-cultural adaptation: The application of international psychological measurement tools requires careful cross-cultural adaptation. Therefore, the conclusions and implications from this study should be limited to the narrow scope of the elite athletes surveyed, and should by no means imply or extrapolate broad generalizability to other sports disciplines or different cohorts of athletes without further empirical evidence.

## Data Availability

The raw data supporting the conclusions of this article will be made available by the authors, without undue reservation.
